# Acute Care of At-Risk Newborns (ACoRN): quantitative and qualitative educational evaluation of the program in a region of China

**DOI:** 10.1186/1472-6920-12-44

**Published:** 2012-06-20

**Authors:** Nalini Singhal, Jocelyn Lockyer, Herta Fidler, Khalid Aziz, Douglas McMillan, Xiangming Qiu, Xiaolu Ma, Lizhong Du, Shoo K Lee

**Affiliations:** 1 Faculty of Medicine, University of Calgary, Calgary, AB, Canada; 2 Faculty of Medicine and Dentistry, University of Alberta, Edmonton, AB, Canada; 3 Faculty of Medicine, Dalhousie University, Halifax, NS, Canada; 4 Faculty of Medicine, University of Toronto, Toronto, ON, Canada; 5 Children’s Hospital of Zhejiang University School of Medicine, Hangzhou, People’s Republic of China

**Keywords:** Newborn care, Newborn stabilization, Life support course, Acute care of at risk newborn, Continuing professional development, Continuing education

## Abstract

**Background:**

The Acute Care of at-Risk Newborns (ACoRN) program was developed in Canada for trained health care providers for the identification and management of newborns who are at-risk and/or become unwell in the first few hours or days after birth. The ACoRN process follows an 8-step framework that enables the evaluation and management of babies irrespective of the experience or expertise of the caregiving individual or team. This study assesses the applicability of the program to Chinese pediatric practitioners.

**Methods:**

Course content and educational materials were translated from English into Chinese by bilingual neonatal practitioners. Confidence and knowledge questionnaires were developed and reviewed for face and content validity by a team of ACoRN instructors. Bilingual Chinese instructors were trained at the tertiary perinatal centre in Hangzhou Zhejiang to deliver the course at 15 level II county hospitals. Participants completed pre- and post-course confidence and knowledge questionnaires and provided feedback through post-course focus groups.

**Results:**

216 physicians and nurses were trained. Confidence and knowledge relating to neonatal stabilization improved significantly following the courses. Participants rated course utility and function between 4.2 and 4.6/5 on all items. Pre/post measures of confidence were significantly correlated with post course knowledge. Focus group data supported the perceived value of the program and recommended course adjustments to include pre-course reading, and increased content related to simulation, communication skills, and management of respiratory illness and jaundice.

**Conclusions:**

ACoRN, a Canadian educational program, appears to be well received by Chinese health care providers and results in improved knowledge and confidence. International program adaptation for use by health care professionals requires structured and systematic evaluation to ensure that the program meets the needs of learners, reflects their learning styles, and can be applied in their setting.

## Background

Worldwide, approximately 4 million babies die in the first 4 weeks after birth with the highest risk being on the first day [1-3]. Almost 99% of these deaths occur in low- and middle-income countries [1,2]. Reductions in neonatal deaths have been relatively limited. Mortality in the first week after birth (the early neonatal period) has shown the least progress, with no measurable change at global levels in the last decade [2,4].

There are evidence-based, cost-effective interventions for improving perinatal care in low-income countries, particularly in rural settings [5-8] which could result in up to a 50% reduction in neonatal mortality [9]. The World Health Organization (WHO) has made substantive efforts to standardize the approach to care of babies worldwide [10]. In addition, the Neonatal Resuscitation Program (NRP) of the American Academy of Pediatrics has been the educational standard internationally for many years [11] and Helping Babies Breathe (HBB) is being assessed for use in resource limited environments [12]. Learner satisfaction; knowledge and skill acquisition and retention; and practical application in urban and rural centers have been evaluated in these programs [12-14]. However, the NRP and HBB programs address only the first few minutes after birth, focusing on resuscitation and the establishment of effective breathing.

The Acute Care of at-Risk Newborns (ACoRN) program [15] was developed in Canada for newborn care providers in the identification and management of newborns who are at-risk and/or become unwell in the first few hours or days after birth. While ACoRN is designed for the Canadian healthcare system, it has been unclear whether ACoRN can be disseminated and sustained in other jurisdictions, nor how acceptable it might be to international learners.

In a promising trend, the People’s Republic of China reduced neonatal mortality by 70% (from 34 to 10 per thousand live births) between 1990 and 2008 [16]. Despite this progress, it is estimated that with a 2008 population of 1.3 billion and a birth rate of 12.14%; approximately 160,000 newborn infants die annually in the first month after birth [16]. After China successfully introduced NRP in Chinese hospitals [17], the next educational question was whether a post-resuscitation stabilization program (e.g., ACoRN) could be effective in China.

The purpose of this study was to assess whether (1) course participants in China perceived the content and program focus useful; (2) the content could be applied to the clinical practice in China and be taught in their institutions; (3) the program increased practitioner self-confidence in managing sick infants; (4) the course improved their knowledge; and (5) the gain in knowledge could be explained by initial knowledge or confidence.

## Methods

### The setting and participants

Funding support from the Canadian Institutes of Health Research and the National Natural Science Foundation of China made it possible for Canadian and Chinese researchers to partner in the dissemination and assessment of the ACoRN program. The Public Health Bureau of Zhejiang Province, along with the Department of Neonatology, Hangzhou Children’s Hospital, Zhejiang University School of Medicine, are committed to the introduction of ACoRN in 15 county hospitals. Zhejiang province in eastern China has a population of 46 million, 2 large cities, 9 regional cities and 36 counties. Each county is served by a county hospital and between 10–20 village clinics. There are about 30,000 births per year in the designated area. Zhejiang has excellent administrative organization and clear lines of communication between levels of care.

The study was conducted in 15 level II county hospitals in South-West Zhejiang province, which is predominantly rural and economically disadvantaged. The total annual births in these hospitals was 22400 (median: 1341; range: 780–3600). There were 4100 (median: 234; range: 120–630) annual nursery admissions. The majority of these deliveries are in a facility. Some are transferred from a lower level facility to a higher level facility for care.

There was an identified need for the ACoRN program for both physicians and nurses. While, almost 100% of the nurses and physicians have participated in an NRP course, there are few opportunities for hands on practice and no policies for continuing assessment of skills or retraining. There is a shortage of nurses in China. Often nurses in special care nurseries will have to take care of 5–6 (or even 8–10 sick babies at night). The ACoRN primary survey (algorithm) enables the nurses to evaluate the baby’s condition quickly. In local hospitals, where most of the medical care is delivered by a general pediatrician with little experience and training in managing sick newborn infants and no medical consultation to support them, physicians require the training in stabilization that ACoRN offers.

### The intervention

The ACoRN program provides a systematic approach to care, using an 8-step framework to help the practitioner systematically gather information, establish priorities and intervene appropriately. The program provides tools to be used for neonatal assessment and the knowledge and skills for clinical management of sick or ‘at-risk’ newborns [15]. The key components include a needs assessment focusing on learners and the organization, a textbook, an interactive workshop, and an evaluation of learners, organization and clinical outcomes. The course addresses neonatal morbidities such as respiratory distress, sepsis, temperature instability, care of Low Birth Weight (LBW) infants, asphyxia and infection and thus goes beyond the immediate few minutes after birth. The course was modified slightly in accordance with Chinese clinical guidelines. An additional module for jaundice was added as well as information about treatment with antibiotics.

ACoRN is designed to be delivered in the same way that other life support courses are delivered, namely that teachers participate in a teach the teacher (TTT) program prior to delivering the course to ensure the course is delivered consistently across all offerings. TTT training focuses on (1) the primary survey for a baby who is sick or at risk leading to a prioritized problem list, (2) algorithmic sequences that address concerns in specific body systems (e.g., respiratory, cardiovascular, and neurological), (3) case-based learning, (4) preparation for neonatal transport, (5) support of the baby, family, and team, and (6) interactive or simulated skill stations for chest X-ray interpretation, jaundice and admission [15].

To ensure that the content of the program was appropriate for practitioners in this area of China, the course text was translated into Mandarin and checked for accuracy by a bilingual member of the ACoRN editorial board. Canadian faculty taught the first few county hospital workshops alongside the participants of the TTT with an expectation that groups of 10–15 (physician and nurses) would be trained in their own communities. The Canadian faculty spent a day with 4 of the trainers adapting some of the content (i.e., jaundice) and delivery to ensure acceptability to the learners.

### Data collection and analysis

#### Quantitative analysis

To evaluate the course, a 16 item (5 point Likert scale, minimum to maximum agreement) questionnaire assessed perceptions about whether participants found the content and program useful and whether the course functioned as designed. This questionnaire also asked participants if they could apply the content in their settings and could teach it to their co-workers (Table 1). The assessment of course utility and function was administered immediately after the completion of the program. A 14 item (5 point) confidence questionnaire (ranging from “not at all” to “always” confident) examined pertinent aspects of infant wellness (Table 2). Three key types of ACoRN clinical practices were distinguished in establishing self-confidence: identifying clinical signs, deciding what action to take, and performing those actions. The self-confidence tool was administered pre- and immediately post-course. To assess knowledge, four scenarios, each describing the condition of an infant, were provided to participants pre- and post-training. For each scenario, they were given 10 options related to potential wellness, diagnoses, and management, and asked whether the response was true or false (for a description of each scenario see Additional file 1, Additional file 2, Additional file 3 and Additional file 4) allowing a maximum score of 40 correct answers.

**Table 1:** Participant perceptions of utility and course function

**MEASURE OF REPORTED AGREEMENT WITH USEFULNESS OF SESSIONS (n = 204)**	**Number of** **responses**	**Mean**	**Standard** **Deviation**
The focus group discussions	179	4.31	0.65
The respiratory and cardiovascular sections	204	4.47	0.64
The neurology, surgery and fluids sections	204	4.42	0.63
The case discussions	200	4.43	0.68
The “admission” station with monitoring	198	4.40	0.68
The temperature and infection sections	202	4.40	0.63
The skills stations	199	4.35	0.66
The “how to teach” pointers	188	4.27	0.71
**MEASURE OF REPORTED AGREEMENT ABOUT COURSE CONTENT (n = 205)**			
The content is presented in a logical order	205	4.47	0.63
The primary survey and sequences are good learning tools	205	4.58	0.62
The primary survey and sequences are clinically useful	205	4.54	0.65
ACoRN promotes communication and a team approach	205	4.39	0.67
The workshop activities help achieve learning goals, generate interest, and promote critical thinking	203	4.52	0.60
The laminated primary survey and sequences are useful learning tools	204	4.50	0.66
I will apply what I learned in my clinical practice	204	4.49	0.64
I will be comfortable teaching this content in my institution	204	4.23	0.75

**Table 2:** Self-assessment of confidence

**Self-Assessment**	**Pre-Test** **(n = 211)**	**Post-Test** **(n = 211)**
**Identifying clinical signs**		
Baby who is sick	3.69 ± 0.91	4.36 ± 0.61*
Baby with respiratory distress	3.83 ± 0.89	4.43 ± 0.63*
Baby with central cyanosis	3.51 ± 0.99	4.42 ± 0.70*
Baby with signs of shock	3.21 ± 0.94	4.05 ± 0.66*
Baby with jitteriness/seizures	3.56 ± 0.97	4.38 ± 0.63*
Baby needs blood glucose check	3.45 ± 1.10	4.43 ± 0.65*
Baby has early signs of infection	3.04 ± 0.88	4.00 ± 0.74*
**Deciding action to take**		
Tests a sick baby needs	3.36 ± 0.90	4.13 ± 0.71*
Treatments a sick baby needs	3.07 ± 0.96	3.95 ± 0.73*
If baby needs ventilation or CPAP	3.27 ± 1.01	4.21 ± 0.68*
Treatment for low glucose	3.34 ± 1.12	4.37 ± 0.77*
**Performing actions**		
Start treatment with IV fluids	3.54 ± 1.03	4.18 ± 0.81*
Keep a stable temperature	3.63 ± 0.98	4.40 ± 0.65*
Speak with parents about baby	3.50 ± 0.87	4.15 ± 0.72*
Total	47.56 ± 10.37	59.21 ± 7.65*

* Compare with pre-test score, p < 0.01.

Descriptive analyses (means, standard deviations) were calculated for each of the items in the ‘course utility and function’ (Table 1) and ‘confidence’ (Table 2) questionnaires. Frequencies were calculated for correct and incorrect answers for the four scenarios. The reliability of the confidence and knowledge measures were tested using Cronbach’s alpha. This test measures the average correlation among all the items. To assess improvement, a paired sample t-test and an effect size calculation (Cohen’s d) were determined for the scores on the confidence and the scenario knowledge questionnaires. An effect size is a way of quantifying the size of the difference between two times of measurement. The relationship between variables measuring: confidence, knowledge, usefulness (of the course), content (of the course), plans to teach the course and applying the learning of the course was investigated using Pearson product–moment correlation coefficient (Pearson r). To determine whether the gain in knowledge on the scenarios could be explained by perceptions of ability to apply course content, ability to teach the course, pre-course knowledge and confidence skills, these variables were entered into a multi-variate linear regression analysis. This test helps to identify which independent variables can best predict an outcome measure. The independent variables of pre confidence and pre knowledge scores were used to assess which best predicted the post knowledge scores.

#### Qualitative analysis

Participants for focus groups were recruited with the assistance of Chinese colleagues, through their contact with the Hangzhou Children’s Hospital. All participants were informed of the purpose of the study and asked to sign a written informed consent which assured confidentiality and sought permission to audio and video tape the group discussions. The focus group questions, developed by members of the ACoRN team, asked both teachers and participants what they liked about the course, to comment on each of the course components (i.e., schedule, manual, primary survey, sequence skill stations and multiple choice questions), and to provide their perceptions about how the course could be improved. Participants were asked whether they currently take care of sick babies and whether any part of the course was challenging to learn and why. Teachers were also asked if they could follow the translation, the acceptability of the course to their learners, how the course might help learners change how they take care of sick babies, and whether any part would be challenging to teach in their hospital.

The interviews were conducted by a fluent English-Chinese speaking Canadian education researcher in a small comfortable meeting room free from outside distractions. Participants sat around a table so they could see and interact with each other. The audio recordings of the interviews were translated and transcribed into English by the group facilitator. All names were changed on the transcripts to ensure anonymity. Transcripts were coded and analyzed so that feedback about the central themes and issues related to all of the core components of the course could be identified and modifications made to the course as needed.

## Results

### Quantitative analysis

A total of 216 physicians and nurses participated in the program.

The participants rated the utility and function of the course highly with a range of means from 4.23 to 4.58. (Table 1). Participants’ total confidence scores increased post course from a mean of 47.6 (s.d. = 10.4) to 59.2 (s.d. = 7.7); effect size d = 1.28. (Table 2) For the confidence measure Cronbach’s alpha was 0.93 for the pre test and 0.94 for the post test indicating high reliability.

The knowledge evaluations indicated that the knowledge scores for each scenario increased between the pre and the post test with a total increase from 31.47 (s.d. = 5.1) to 34.7 (s.d. = 3.5) out of 40; effect size d = 0.77. While this increase in scores is only about 10%, it is noted that the increase was greater for the three scenarios in which there were abnormal findings compared to scenario D which profiled a well term infant. For the pre knowledge measure, the Cronbach’s alpha was 0.62 and post measure Cronbach’s alpha was 0.58 (Table 3 and for pre and post scores see Additional file 1, Additional file 2, Additional file 3 and Additional file 4).

**Table 3:** Scenarios for evaluation of knowledge

**Scenarios**	**Pre-Test Mean** **Score (n = 210)**	**Post-Test Mean** **Score (n = 210)**	**Effect Size**
Scenario A; A term infant with respiratory distress	8.28 ± 1.58	9.04 ± 1.14*	0.55
Scenario B: A 2-hour old 32 week preterm infant with respiratory distress	7.90 ± 1.83	9.18 ± 1.05*	0.87
Scenario C: A 4-day old baby with jaundice, fever and a seizure	8.39 ± 1.53	9.56 ± 0.81*	0.96
Scenario D: A well term infant after intubation and suctioning for meconium-stained amniotic fluid	6.95 ± 2.10	7.10 ± 1.93	Not significant
Total (maximum 40)	31.37 ± 5.12	34.74 ± 3.53*	0.77

* Compare with pre-test score, p < 0.01.

The correlation analysis revealed that all of the significant correlations were positive and ranged from small to large. Measures of usefulness of the course, content of the course, applying what was learned and comfort with teaching the course were highly correlated sharing from 34% of the variance (between assessment of the content and assessment of the usefulness of the course) to 62% of the variance between the content and applying the course. Pre measure of confidence and post measure of confidence were significantly correlated with most measures of knowledge; although, a number of these correlations were small (Table 4).

**Table 4:** Correlations of pre and post measures

	**1**	**2**	**3**	**4**	**5**	**6**	**7**	**8**	**9**	**10**	**11**	**12**	**13**	**14**
1 Pre confidence	1	.586**	.303**	.280**	.383**	.232**	.395**	.155*	NS	.162*	NS	.148*	NS	NS
2 Post confidence		1	.230**	.323**	.235**	.234**	.205**	.207**	NS	.140*	NS	NS	NS	NS
3 Pre Scenario A			1	.330**	.471**	.155*	.319**	NS	NS	.142*	NS	NS	NS	NS
4 Post Scenario A				1	.193**	.335**	.172*	.236**	NS	NS	NS	NS	NS	NS
5 Pre Scenario B					1	.342**	.645**	.221**	.173*	NS	NS	.145*	NS	NS
6 Post Scenario B						1	.227**	.507**	NS	NS	NS	NS	NS	NS
7 Pre Scenario C							1	NS	.220**	NS	NS	.154*	NS	NS
8 Post Scenario C								1	NS	NS	NS	NS	NS	NS
9 Pre Scenario D									1	.497**	NS	NS	NS	NS
10 Post Scenario D										1	NS	NS	NS	NS
11 Total Usefulness											1	.672**	.527**	.628**
12 Total Content												1	.589**	.795**
13 Teaching													1	.643**
14 Applying Learning														1

NS = Not significant; **. Correlation is significant at the 0.01 level (2-tailed); *. Correlation is significant at the 0.05 level (2-tailed).

A backward linear regression analysis was used to test if pre knowledge and pre comfort could explain post knowledge scores. The results of the regression indicated that while both variables were significant and explained 26% of the variance (R2 = 0.26, F(2,207) = 36.514, p < 0.01); the majority of the variance was accounted for by pre course knowledge (β = .440, p < .001) with pre comfort scores (β = 138, p < 0.05) accounting for less of the variance (Table 5).

**Table 5:** Linear regression analysis

**Variable**	**B**	**SE B.**	***β***
Pre Knowledge	.302	.045	.440**
Pre Comfort	.048	.022	.138*
R^2^		.261	
F for change in R^2^		36.514**	

Summary of Backward Regression Analysis Predicting Post Knowledge Scores

*p < .05, **p < .00.

### Qualitative analysis

The focus group held with the 11 teachers provided information about several aspects of the course that they liked. They reported that the course clearly outlined the process and taught a rigorous systematic approach which included all aspects of at-risk and unwell newborns. This made the course suitable for beginners. Specifically, the teachers found the content of the textbook well designed and the primary survey good. They noted that teaching about X-rays was practical and could easily be used by local physicians. The teaching process did not require a theoretical base or high prior skills. They felt that the two days of training was very good.

The teachers recommended some modifications to the course. They suggested that course expectations for learners should be more clearly outlined. They noted that implementation would require them to consider local conditions such as the clinical resources, skill level, and equipment which should be taken into account. Similarly, cases needed to be re-designed for the local setting with some modification to reduce their complexity. They identified the need for additional work on the jaundice skill station and feeding for the at risk infants. The teachers commented on the amount of time it would take to go through the sequences in a very busy clinical practice. They were concerned that changing the practice of providers would be difficult, especially for older clinicians. (for participant quotes see Additional file 5)

Two focus groups were held for learners with a total of 19 participants. The learners were very positive about the course. They stated that the content was clear and concise with a logical and systematic order that made it easy to understand. They found it suitable for clinical practice and holistic in its coverage of at-risk newborns. Teachers were seen as knowledgeable, interacting with participants and willing to answer questions. Particular elements that were identified as strong included the text book and its clear contents, the use of the primary survey to introduce proper procedures and the usefulness of the sequence skill station. The learners felt the workshop would be useful to rural physicians and that they could teach the material.

Some challenges with the course were described by the learners. While the primary survey (algorithm) was seen as good, they recommended modifications so that it would be more useful and suitable for the Chinese environment. They encouraged adding more information about the respiratory system and jaundice to the course and to the textbook. Last, learners suggested that clinical simulations would be helpful.

Learners also recommended pre-course material including a clear workshop schedule and textbook so they could prepare for the course. They noted that attention also needed to be paid to post course activities to reinforce the content to prevent participants from going back to their old ways. (for participant quotes see Additional file 5)

## Discussion

This is an evaluation of the ACoRN course in a setting very different from the one in which it was initially developed. As noted earlier, the partners in China had identified a need for the program. There were shortages of nurses, physicians were not trained to care for ill newborns, and usable algorithms did not exist to evaluate babies quickly. The team of 3 Canadians and 4 Chinese teachers worked together to review the course material in a step by step procedure. They wanted to ensure that both the material and the delivery were appropriate before the program was delivered. These discussions led to modification of content, namely information about managing jaundice was added to the course. Teaching was also modified. In China, teaching is traditionally didactic and teacher directed. In Canada, the program would be handled through a facilitative process in which learners would be encouraged to provide their opinions and ideas and work towards group solutions. The discussion between Chinese and Canadian teachers led to a teaching structure in which the teaching became more directed than in Canada (less facilitative) with the teachers providing explicit information about patient management. We presented the skill stations and demonstrated the interactive participatory method of learning. The students very quickly learnt how to conduct them and were successfully engaged in the participatory learning. We speculate that in settings where traditional didactic methods of teaching are practiced exposure to participatory methods will be acceptable and may lead to acquisition and sustainability of knowledge. The initial evaluation results of these modifications to the ACoRN course in this setting are encouraging and suggest that the program with modifications could be used in China.

Participants told us that the content and focus of the course were useful. Further, participants believed they could apply what they learned in their own clinical practices. The course functioned reasonably well with teachers reporting they would be comfortable teaching the content and learners demonstrating an increase in knowledge and self- confidence. For both self-confidence and knowledge, the effect size of the increase in scores could be considered to be ‘large’. The variance in the scores at the post-test was primarily explained by the pre course knowledge scores; while the pre-comfort score did make a statistically unique contribution to the equation it added very little to the variance.

By taking both a quantitative and qualitative approach to data collection, we were able to assess change and improvement in scores as well as obtaining critical information to modify the course. The focus groups provided us with additional insights about participants’ desires to prepare for the learning experience, areas which needed to be expanded, and cases which were inappropriate for learners in China.

One of the interesting outcomes of this study was the preference of Chinese learners for case-based learning and simulation. Research on learning through simulation suggests that when it is well planned, it has benefits, particularly in complex situations involving teams [18]. This is an area of ACoRN that undoubtedly needs further examination and development to incorporate deliberate practice and feedback into its design in a more rigorous way to maximize outcomes [19].

The results of this study do speak to the generalizability of the ACoRN Primary Survey and Sequences, presumably because of the generic nature of newborn diseases. Gaps, however, were identified in learning objectives, in particular, the need to add neonatal jaundice to the list of conditions addressed.

This evaluation is an initial assessment of the program. As health professions educators have noted, a comprehensive assessment of educational outcomes should include a hierarchy that considers participant numbers, satisfaction, learning, competence and performance; patient health; and community health [20]. This study addresses the first four-components. The next stages of assessment will include examinations of patient outcomes drawing on patient data collected prior to and after the educational interventions. A significant challenge and therefore limitation of this study was the need to develop evaluation instruments for both confidence and knowledge in the absence of a “gold standard”. The questionnaires did stand up well, with good correlations between domains, as well as support from qualitative analysis. These data are helpful in establishing the value of ACoRN evaluations.

Educational and other strategies continue to be important in the challenge of improving neonatal outcomes. As a recent Cochrane review noted, it continues to be important to have skilled delivery and facility-based services for newborn care as well as community-based care [8]. The applicability of the ACoRN education program to two healthcare systems and multiple professions is the first step in determining whether it will improve outcomes for babies and families.

## Conclusion

ACoRN, a Canadian educational program, was developed for a Canadian population of health care providers. Through a collaborative effort whereby Canadian and Chinese neonatologists worked together, the ACoRN program was modified both to add content and to change the teaching strategies in order to improve the program’s utility and acceptability. While data are preliminary, the program appeared to be well received by Chinese health care providers and resulted in improved knowledge and confidence. Programs developed in one setting can be modified and successfully adapted for a different setting.

### Ethical approval

University of Calgary Conjoint Health Ethics Review Board.

## Abbreviations

ACoRN, Acute Care of at-Risk Newborn; HBB, Helping Babies Breathe, a program of the American Academy of Pediatrics; NRP, Neonatal Resuscitation, a program of the American Academy of Pediatrics.

## Competing interests

The authors declare that they have no competing interests.

## Authors’ contributions

NS was PI on the grant application, oversaw all aspects of the study in Canada and China, and drafted the manuscript. JL was a co-investigator on the grant, provided critical input on instruments and data analysis, oversaw the analysis, drafted the manuscript and managed manuscript revisions. HF was research associate on the study, handled the analysis, created tables, and provided critical input into the manuscript. KA was a co-investigator on the grant, collaborated on the adaptation of the course, provided critical input on instruments and data analysis, and provided critical input into the manuscript drafts. DM was a co-investigator on the study, collaborated on the adaptation of the course, provided critical input on instruments and data analysis, and provided critical input into the manuscript drafts. XQ was a research associate on the study, conducted the focus groups, analyzed the qualitative data, and provided critical input into manuscript drafts. XM collaborated on the adaptation of the course for China, co-managed the educational programming and data collection in China, and provided critical input into manuscript drafts. LD was project lead for China, collaborated on the adaptation of the course for China, co-managed the educational programming and data collection in China, and provided critical input into manuscript drafts. SL was a co-investigator on the grant and provided critical input into manuscript drafts. All authors read and approved the final manuscript.

## Authors’ information

Dr Singhal is Professor, Department of Pediatrics, Faculty of Medicine, University of Calgary, Calgary, Alberta, Canada

Dr Lockyer is Professor, Department of Community Health Sciences and Senior Associate Dean--Education, Faculty of Medicine, University of Calgary, Calgary, Alberta

Ms Fidler is Research Associate, Office of Continuing Medical Education and Professional Development, Faculty of Medicine, University of Calgary, Calgary, Alberta, Canada

Dr Aziz is Associate Professor, Faculty of Medicine and Dentistry, University of Alberta, Edmonton, Alberta, Canada

Dr McMillan is Professor Department of Pediatrics, Faculty of Medicine, Dalhousie University, Halifax, Nova Scotia, Canada

Dr Qiu is Assistant Professor of Paediatrics, Faculty of Medicine, University of Toronto, Toronto, Ontario Canada

Dr Ma is Assistant Professor, Pediatrics, Children’s Hospital of Zhejiang University School of Medicine, Hangzhou, People’s Republic of China

Dr Du is Professor, Pediatrics, Children’s Hospital of Zhejiang University School of Medicine, Hangzhou, People’s Republic of China

Dr Lee is Professor, Departments of Paediatrics, Obstetrics & Gynecology, and Public Health, Faculty of Medicine, University of Toronto, Toronto, Ontario, Canada

## Pre-publication history

The pre-publication history for this paper can be accessed here:

http://www.biomedcentral.com/1472-6920/12/44/prepub

## Supplementary Material

Additional file 1 **Scenario A results.** Participants were asked to assess a 2 hour old full term baby from the maternity unit. The baby looks pale and has blue hands and feet. He is making a noise with every breath when he breathes out. His breathing rate is 40 per minute. His pulse rate is 120 per minute. His axillary temperature is 35.8 Celsius. He does not wake up when you examine him. Click here for file

Additional file 2 **Scenario B results.** Participants were asked to assess a 32 week gestation, 1.8 kg baby is admitted to the nursery at two hours of age. The baby has breathing difficulty and looks pale and blue despite receiving 100% oxygen by facemask. His breathing rate is 50 per minute and his pulse rate is 150 per minute. His axillary temperature is 36.8 Celsius. Click here for file

Additional file 3 **Scenario C results.** Participants were asked to assess a full term baby admitted at 4 days of age with jaundice, a fever, and a seizure. The baby is breathing easily at 40 breaths per minute with aheart rate of 120 per minute. His axillary temperature is 38 Celsius. He is no longer seizing but does not wake up when examined. The transcutaneous bilirubin level is >305 micromol/l(>18 mg/dl). Click here for file

Additional file 4 **Scenario D results.** Participants were asked to assess a full term baby who required intubation and suctioning for meconium-stained amniotic fluid. At 10 minutes of age, the baby appears pink and is breathing easily with a respiratory rate of 40 breaths for minute. The pulse rate is 120 beats per minute. Capillary refill time is 3 seconds. The baby is alert and active. Click here for file

Additional file 5 **Qualitative data from focus groups.** Direct quotes from instructors and learners are documented in this file. Click here for file
