# Supplementary material for: Acute Care of At-Risk Newborns (ACoRN): quantitative and qualitative educational evaluation of the program in a region of China
Source: BMC Med Educ. 2012 Jun 20;12:44. doi: 10.1186/1472-6920-12-44 (PMC3437201; doi:10.1186/1472-6920-12-44)
Supplement: Additional file 1 — Scenario A results. Participants were asked to assess a 2 hour old full term baby from the maternity unit. The baby looks pale and has blue hands and feet. He is making a noise with every breath when he breathes out. His breathing rate is 40 per minute. His pulse rate is 120 per minute. His axillary temperature is 35.8 Celsius. He does not wake up when you examine him. [file 1472-6920-12-44-S1.pdf]

Scenario A:

You are asked to assess a 2 hour old full term baby from the maternity unit. You see the baby and find that the baby looks pale and has blue hands and feet. He is making a noise with every breath when he breathes out. His breathing rate is 40 per minute. His pulse rate is 120 per minute. His axillary temperature is 35.8 Celsius. He does not wake up when you examine him.

**Total N=210**	**Pre test responses**		**Post test responses**	
	**Correct (%)**	**Incorrect (%)**	**Correct (%)**	**Incorrect (%)**
1. This baby is unwell.	199 (94.8)	7 (3.3)	197 (93.8)	10 (4.8)
2. Blue hands and feet are a normal finding at two hours of age.	187 (89.0)	15 (7.1)	187 (89.0)	23 (11.0)
3. Noisy breathing with each breath is normal at two hours of age.	189 (90.0)	13 (6.2)	191 (91.0)	16 (7.6)
4. A breathing rate of 40 per minute is normal at two hours of age.	178 (84.8)	29 (13.8)	201 (95.7)	9 (4.3)
5. A heart rate of 120 per minute in a resting baby is normal.	191 (91.0)	16 (7.6)	204 (97.1)	4 (1.9)
6. An axillary temperature of 35.8	164 (78.1)	42 (20.0)	195 (92.9)	14 (6.7)

Celsius is a normal finding.				
7. This baby needs a check of its blood glucose.	182 (86.7)	11 (5.2)	200 (95.2)	7 (3.3)
8. This baby can remain with its mother in the maternity unit.	181 (86.2)	22 (10.5)	177 (84.3)	27 (12.9)
9. This baby may have an infection.	150 (71.4)	27 (12.9)	193 (91.9)	11 (5.2)
10. This baby needs surfactant.	118 (56.2)	36 (17.1)	154 (73.3)	19 (9.0)
