# Supplementary material for: Acute Care of At-Risk Newborns (ACoRN): quantitative and qualitative educational evaluation of the program in a region of China
Source: BMC Med Educ. 2012 Jun 20;12:44. doi: 10.1186/1472-6920-12-44 (PMC3437201; doi:10.1186/1472-6920-12-44)
Supplement: Additional file 2 — Scenario B results. Participants were asked to assess a 32 week gestation, 1.8 kg baby is admitted to the nursery at two hours of age. The baby has breathing difficulty and looks pale and blue despite receiving 100% oxygen by facemask. His breathing rate is 50 per minute and his pulse rate is 150 per minute. His axillary temperature is 36.8 Celsius. [file 1472-6920-12-44-S2.pdf]

Scenario B:

A 32 week gestation, 1.8 kg baby is admitted to your nursery at two hours of age. The baby has breathing difficulty and looks pale and blue despite receiving 100% oxygen by facemask. His breathing rate is 50 per minute and his pulse rate is 150 per minute. His axillary temperature is 36.8 Celsius.

**Total N=210**	**Pre test responses**		**Post test responses**	
	**Correct (%)**	**Incorrect (%)**	**Correct (%)**	**Incorrect (%)**
1. This baby is unwell.	197 (93.8)	9 (4.3)	204 (97.1)	6 (2.9)
2. This baby needs to be supported with ventilation or CPAP.	162 (77.1)	30 (14.3)	171 (81.4)	35 (16.7)
3. A cuff blood pressure of 25 mm Hg mean is normal for this baby.	139 (66.2)	18 (8.6)	195 (92.9)	9 (4.3)
4. A capillary refill time of 5 seconds is normal for this baby.	127 (60.5)	13 (6.2)	200 (95.2)	6 (2.9)
5. A heart rate of 150 per minute is normal for this baby.	149 (71.0)	54 (25.7)	191 (91.0)	18 (8.6)
6. An axillary temperature of 36.8	190 (90.5)	14 (6.7)	202 (96.2)	7 (3.3)

Celsius is a normal finding.				
7. A blood glucose of 1.4 mmol/l (25 mg/dl) is normal for this baby.	180 (85.7)	11 (5.2)	204 (97.1)	3 (1.4)
8. This baby needs intravenous fluids.	188 (89.5)	9 (4.3)	197 (93.8)	8 (3.8)
9. This baby may have an infection.	167 (79.5)	17 (8.1)	203 (96.7)	1 (.5)
10. This baby needs surfactant.	160 (76.2)	13 (6.2)	161 (76.7)	33 (15.7)
