# Supplementary material for: Acute Care of At-Risk Newborns (ACoRN): quantitative and qualitative educational evaluation of the program in a region of China
Source: BMC Med Educ. 2012 Jun 20;12:44. doi: 10.1186/1472-6920-12-44 (PMC3437201; doi:10.1186/1472-6920-12-44)
Supplement: Additional file 3 — Scenario C results. Participants were asked to assess a full term baby admitted at 4 days of age with jaundice, a fever, and a seizure. The baby is breathing easily at 40 breaths per minute with aheart rate of 120 per minute. His axillary temperature is 38 Celsius. He is no longer seizing but does not wake up when examined. The transcutaneous bilirubin level is >305 micromol/l(>18 mg/dl). [file 1472-6920-12-44-S3.pdf]

Scenario C

A full term baby is admitted at 4 days of age with jaundice, a fever, and a seizure. The baby is breathing easily at 40 breaths per minute with a heart rate of 120 per minute. His axillary temperature is 38 Celsius. He is no longer seizing but does not wake up when you examine him. The transcutaneous bilirubin level is >305 micromol/l(>18 mg/dl).

**Total N=209**	**Pre test responses**		**Post test responses**	
	**Correct (%)**	**Incorrect (%)**	**Correct (%)**	**Incorrect (%)**
1. This baby is unwell.	206 (98.6)	3 (1.4)	205 (98.1)	4 (1.9)
2. This baby needs to be supported with ventilation.	193 (92.3)	7 (3.3)	202 (96.7)	6 (2.9)
3. A cuff blood pressure of 25 mm Hg mean is normal for this baby.	126 (60.3)	29 (13.9)	195 (93.3)	11 (5.3)
4. A capillary refill time of 5 seconds is normal for this baby.	123 (58.9)	24 (11.5)	200 (95.7)	3 (1.4)
5. A heart rate of 150 per minute is normal for this baby.	141 (67.5)	59 (28.2)	188 (90.0)	21 (10.0)
6. An axillary	200 (95.7)	7 (3.3)	207 (99.0)	2 (1.0)

temperature of 38 Celsius is a normal finding.				
7. A blood glucose of 1.4 mmol/l (25 mg/dl) is normal for this baby.	177 (84.7)	13 (6.2)	205 (98.1)	3 (1.4)
8. This baby needs intravenous fluids	188 (90.0)	12 (5.7)	195 (93.3)	11 (5.3)
9. This baby may have an infection.	196 (93.8)	7 (3.3)	201 (96.2)	3 (1.4)
10. This baby needs phototherapy.	203 (97.1)	1 (.5)	201 (96.2)	5 (2.4)
