# Supplementary material for: Acute Care of At-Risk Newborns (ACoRN): quantitative and qualitative educational evaluation of the program in a region of China
Source: BMC Med Educ. 2012 Jun 20;12:44. doi: 10.1186/1472-6920-12-44 (PMC3437201; doi:10.1186/1472-6920-12-44)
Supplement: Additional file 4 — Scenario D results. Participants were asked to assess a full term baby who required intubation and suctioning for meconium-stained amniotic fluid. At 10 minutes of age, the baby appears pink and is breathing easily with a respiratory rate of 40 breaths for minute. The pulse rate is 120 beats per minute. Capillary refill time is 3 seconds. The baby is alert and active. [file 1472-6920-12-44-S4.pdf]

Scenario D

A full term baby required intubation and suctioning for meconium-stained amniotic fluid. At 10 minutes of age, the baby appears pink and is breathing easily with a respiratory rate of 40 breaths for minute. The pulse rate is 120 beats per minute. Capillary refill time is 3 seconds. The baby is alert and active.

**Total N =207**	**Pre test responses**		**Post test responses**	
	**Correct (%)**	**Incorrect (%)**	**Correct (%)**	**Incorrect (%)**
1. This baby is unwell.	119 (57.5)	85 (41.7)	99 (47.8)	107 (51.9)
2. This baby can be breastfed.	150 (72.5)	55 (26.8)	155 (74.9)	52 (25.1)
3. This baby can stay with its mother.	138 (66.7)	66 (32.4)	143 (69.1)	62 (30.2)
4. A capillary refill time of 3 seconds is normal for this baby.	164 (79.2)	41 (20.0)	198 (95.7)	8 (3.9)
5. A heart rate of 120 per minute is normal for this baby.	195 (94.2)	11 (5.3)	207 (100.0)	0
6. An axillary temperature of 36.5 Celsius would be a normal finding.	201 (97.1)	5 (2.4)	204 (98.6)	3 (1.4)

7. This baby needs to have its blood glucose checked.	77 (37.2)	128 (62.4)	48 (23.2)	158 (76.7)
8. This baby needs intravenous fluids.	138 (66.7)	67 (32.7)	140 (67.6)	66 (32.0)
9. This baby needs antibiotics.	67 (32.4)	140 (67.6)	84 (40.6)	123 (59.4)
10. This baby needs phototherapy.	189 (91.3)	18 (8.7)	192 (92.8)	15 (7.2)
