# Supplementary material for: Acute Care of At-Risk Newborns (ACoRN): quantitative and qualitative educational evaluation of the program in a region of China
Source: BMC Med Educ. 2012 Jun 20;12:44. doi: 10.1186/1472-6920-12-44 (PMC3437201; doi:10.1186/1472-6920-12-44)
Supplement: Additional file 5 — Qualitative data from focus groups. Direct quotes from instructors and learners are documented in this file. [file 1472-6920-12-44-S5.pdf]

Qualitative data from focus groups

**Course Content and Assessment Tools**	**Instructor Themes**	**Instructor Representative quote(s)**	**Learner Themes**	**Learner Representative quote(s)**
Course manual	Satisfied with the primary survey systematic approach	"The ACoRN textbook contents are well designed and the sequences are useful. It will be helpful for beginners, because it provides steps for clinical practice."	Useful textbook	"The textbook is good, and the content is clear. It is suitable for our local situations"<br><br>"The primary survey taught in this workshop forces everybody, no matter if they are

				experienced, to follow the same steps for treatment. No one can miss any of the steps for consideration, and this is good.”
Mega cases	-Better preparation needed for both instructors & learners<br>-need to be more realistic, practical and suitable for Chinese learners.	“If it’s well-designed, it should be very good. It shouldn’t be carried out in a large open space as in our case. It should be in a simulated environment.”<br><br>“These instructors haven’t become familiar with our environment. It should be well-prepared and well-		

		designed for it.”<br><br>“I felt today’s mega-cases were purposefully designed to be complicated. It appears that they purposefully presented cases with situations in which infants had all types of problems simultaneously…when you have a complicated case, it also requires the ability to process all the information at once, making teaching more difficult and challenging. Students can get lost and get into a loop without a clear direction to follow…In today’s situation it		

		seems that none of us were prepared for what was expected. Consequently, the results were not ideal."<br><br>"The mega-cases need more adequate preparation and need to be more realistic and practical for learners here."		
Clinical information	The sequence skill stations provide useful cases to learn from	"I felt the best part is the X-ray. If we teach the local physicians, those are very practical and can be easily absorbed. The instruction was very good, demonstrative, and the important points were demonstrated well. The local	Need clinical case as an example for practice	"It is not about how fast they taught us, it is how to provide concrete cases for us so we know how to follow the

		physicians will benefit from it.”		sequences.”
**Course Processes**				
Teaching methods	-The thought process was valuable<br>-Need to be improved	“The approach is good because it allows learners to participate in the learning process so everyone has a chance to practice.” “It requires the instructor to understand the level of the students, and know exactly what to teach and what to expect from the students. The instructor should be able to give the proper guidance and correction at the moment when it’s needed. So as instructors, we have to be very familiar with	-Able to interact with the instructors directly<br><br>-Needs concrete cases to learn from	“The teaching method was good. We were able to interact with the instructors directly, so it’s beneficial that way.”<br><br>“A clinical simulation would really help us learn the course content better.”

		the teaching material, then we can guide and provide clear directions for the students to follow.”<br><br>“During teaching, if students are learning, you need to make sure to let them know clearly beforehand teaching sequences and what they are expected to. It shouldn’t be the case that 4 or 5 students all do the same thing at the same time and ignore other things (mega-case). There should be a facilitator as a leader coordinating the process to make sure everybody knows exactly their		

		responsibilities in terms of what to expect and what to do. Otherwise, it would be chaotic and ineffective.”		
Pace of course			Too fast & feel being rushed	“The speed of the course is too fast, especially in between the sessions. It seemed to me we were rushed to follow the next session without having time to ask questions and digest the materials. There wasn’t

				enough time to cognitively process the course content and to formulate relevant questions. For example, we did not have time to ask questions in between the sequences of the primary survey. It was too rushed and it should be introduced and explained

				more adequately and slowly.”
Other feedback about course and its directions	Concerns about the practical aspects for the local learners	“If you attempt to implement the ACoRN program in the local hospitals, you will first need to know the local conditions such as the clinical resources, skill level, and equipment; also you need to design cases that are suitable for the local conditions. Therefore, you must adjust the teaching approach to fit local needs.”<br><br>“For the local physicians, it might be difficult to adapt. It probably will take a	Topics need to be covered for local hospitals	“How to communicate with patient families should be covered, especially the proper skills and ways of communicating”<br><br>“Issues need to be communicated with parents, such as transportation, blood

		longer time for them to learn."<br><br>"For the local physicians, it might take more time to get used to the procedure and thinking process."		transfusion, and the possible consequences of procedures."<br><br>"Jaundice which is rather relevant to our clinical practices should be included in the textbook."
	Better preparation needed for both instructors & learners	"In today's situation it seems that none of us were prepared for what was expected. Consequently, the results were not ideal."	Need better preparation	"The organization of the workshop needs to be improved.

				There should be at least 3 days for this workshop. We were not well informed of the schedule of the course and this created unnecessary inconvenience."<br><br>"the laminates should be introduced in a clearer fashion, specifically what each

				laminate is about, therefore new students can have a better concept of the purpose of each laminate.”
	How to evaluate the program	“How do we evaluate how much the learners have learned and whether there is a change in their practice. It is not only the evaluation of this, as a matter of fact, the evaluation of the integrated thinking process, clinical practice and the process of dealing with cases.”	Difficulties in implementation	“The challenge would be how to apply what we have learned in practice. It would be difficult to correct old habits and replace them with new ones. Also,

				conditions of different clinics can vary so much, it would be difficult to implement them.”
